# Supplementary material for: Illustrating Implications of Misaligned Causal Questions and Statistics in Settings With Competing Events and Interest in Treatment Mechanisms
Source: Stat Med. 2026 Apr 24;45:e70535. doi: 10.1002/sim.70535 (PMC13109003; doi:10.1002/sim.70535)
Supplement: Supplementary file 1 — Supplemental Table 1. Ranges of parameter values for logistic models (20) and (21) in data generating scenarios for Figures 3, 4, and Supplemental Figure 1. The intercept θ0 for the model (20) was fixed at −1 in all figures. The intercept β0 for the model (21) was set to −1 for all panels in Figures 3 and 4 (where D is non‐rare), and to −9 (left panels) and −6 (right panels) in Supplemental Figure 1 (where D is rare). Supplemental Table 2. Specifications of the logistic model coefficients used in simulation study, Section 4.3. In all scenarios, (pL,pU,θ0,θ1,θ2,θ3) were fixed at (0.1,0.5,−1,−2,1,3). Supplemental Table 3. Simulation‐based evaluation of the variance Var(SDE^obsaD=1). Expectations in the variance calculations were taken relative to the distribution over simulation runs. Supplemental Table 4. Regression coefficients for other‐cause death by the pooled logistic model fitted to the prostate cancer data used in Section 5. Supplemental Figure 1. Illustration of estimand error when the actual causal target is a separable direct effect but the ostensible causal target is a controlled direct effect in the scenarios where D is rare (i.e., under the parameters given in Supplemental Table 1 where Pr(D=1|A=a,L=l,U=u)<10% for all (a,l,u)). Each dot plots the value of the actual versus the ostensible causal target for a combination of the parameter values. [file SIM-45-0-s001.pdf]

Supplementary Materials for "Illustrating implications of misaligned causal questions and statistics in settings with competing events and interest in treatment mechanisms" by Takuya Kawahara, Sean McGrath and Jessica G. Young

Section S1. Derivation of the formulas for non-identification errors

In the Appendix A of the main text, we showed an expression for the *estimand error*,

$$\begin{aligned}\mathrm{CDE}_0 - \mathrm{SDE}_0^{a_D} = &\{\psi_0(a=1,d=0) - \psi_0(a=0,d=0)\} \\ &- \{\psi_0(a_Y=1,a_D) - \psi_0(a_Y=0,a_D)\},\end{aligned} \tag{S1}$$

in terms of the joint distribution of (U, L, A, D, Y),

$$\begin{aligned}\mathrm{CDE}_0 - \mathrm{SDE}_0^{a_D} =&\{\mu_0(1,1,1) - \mu_0(0,1,1)\}\pi_0(a_D,1,1)p_Lp_U \\ &+ \{\mu_0(1,1,0) - \mu_0(0,1,0)\}\pi_0(a_D,1,0)p_L(1-p_U) \\ &+ \{\mu_0(1,0,1) - \mu_0(0,0,1)\}\pi_0(a_D,0,1)(1-p_L)p_U \\ &+ \{\mu_0(1,0,0) - \mu_0(0,0,0)\}\pi_0(a_D,0,0)(1-p_L)(1-p_U).\end{aligned} \tag{S2}$$

Similarly, we showed an expression for the *non-identification error* in any estimator for the controlled direct effect,

$$\begin{aligned}\{\mathrm{CDE}_{obs} - \mathrm{CDE}_0\} = &\{\tilde{\psi}(a=1,d=0) - \tilde{\psi}(a=0,d=0)\} \\ &- \{\psi_0(a=1,d=0) - \psi_0(a=0,d=0)\},\end{aligned} \tag{S3}$$

as

$$
\begin{aligned}
&\{\mathrm{CDE}_{obs} - \mathrm{CDE}_0\} \\
&= - \frac{\pi_0(1,1,1) - \pi_0(1,1,0)}{\{1-\pi_0(1,1,1)\}p_U + \{1-\pi_0(1,1,0)\}(1-p_U)} \{\mu_0(1,1,1) - \mu_0(1,1,0)\} p_L p_U (1-p_U) \\
&\quad - \frac{\pi_0(1,0,1) - \pi_0(1,0,0)}{\{1-\pi_0(1,0,1)\}p_U + \{1-\pi_0(1,0,0)\}(1-p_U)} \{\mu_0(1,0,1) - \mu_0(1,0,0)\} (1-p_L) p_U (1-p_U) \\
&\quad + \frac{\pi_0(0,1,1) - \pi_0(0,1,0)}{\{1-\pi_0(0,1,1)\}p_U + \{1-\pi_0(0,1,0)\}(1-p_U)} \{\mu_0(0,1,1) - \mu_0(0,1,0)\} p_L p_U (1-p_U) \\
&\quad + \frac{\pi_0(0,0,1) - \pi_0(0,0,0)}{\{1-\pi_0(0,0,1)\}p_U + \{1-\pi_0(0,0,0)\}(1-p_U)} \{\mu_0(0,0,1) - \mu_0(0,0,0)\} (1-p_L) p_U (1-p_U)
\end{aligned} \tag{S4}
$$

and for the non-identification error (S5) in any estimator for the separable direct effects,

$$
\begin{aligned}
\{\mathrm{SDE}^{a_D}_{obs} - \mathrm{SDE}^{a_D}_0\} = \{&\tilde{\psi}(a_Y = 1, a_D) - \tilde{\psi}(a_Y = 0, a_D)\} \\
&- \{\psi_0(a_Y = 1, a_D) - \psi_0(a_Y = 0, a_D)\},
\end{aligned} \tag{S5}
$$

as

$$
\begin{aligned}
&\{\mathrm{SDE}^{a_D}_{obs} - \mathrm{SDE}^{a_D}_0\} \\
&= \frac{\{1-\pi_0(1,1,1)\}\{1-\pi_0(a_D,1,0)\} - \{1-\pi_0(a_D,1,1)\}\{1-\pi_0(1,1,0)\}}{\{1-\pi_0(1,1,1)\}p_U + \{1-\pi_0(1,1,0)\}(1-p_U)} \\
&\qquad \times \{\mu_0(1,1,1) - \mu_0(1,1,0)\} p_L p_U (1-p_U) \\
&\quad + \frac{\{1-\pi_0(1,0,1)\}\{1-\pi_0(a_D,0,0)\} - \{1-\pi_0(a_D,0,1)\}\{1-\pi_0(1,0,0)\}}{\{1-\pi_0(1,0,1)\}p_U + \{1-\pi_0(1,0,0)\}(1-p_U)} \\
&\qquad \times \{\mu_0(1,0,1) - \mu_0(1,0,0)\} (1-p_L) p_U (1-p_U) \\
&\quad - \frac{\{1-\pi_0(0,1,1)\}\{1-\pi_0(a_D,1,0)\} - \{1-\pi_0(a_D,1,1)\}\{1-\pi_0(0,1,0)\}}{\{1-\pi_0(0,1,1)\}p_U + \{1-\pi_0(0,1,0)\}(1-p_U)} \\
&\qquad \times \{\mu_0(0,1,1) - \mu_0(0,1,0)\} p_L p_U (1-p_U) \\
&\quad - \frac{\{1-\pi_0(0,0,1)\}\{1-\pi_0(a_D,0,0)\} - \{1-\pi_0(a_D,0,1)\}\{1-\pi_0(0,0,0)\}}{\{1-\pi_0(0,0,1)\}p_U + \{1-\pi_0(0,0,0)\}(1-p_U)} \\
&\qquad \times \{\mu_0(0,0,1) - \mu_0(0,0,0)\} (1-p_L) p_U (1-p_U).
\end{aligned} \tag{S6}
$$

In the subsections below, we derive (S4) and (S6). We show the approximations of the estimand error under rare-disease assumptions in Section S4.

Derivation of (S4)

By definition, the non-identification error is given by

$$\begin{aligned}\text{CDE}_{obs} - \text{CDE}_0 = &\{\tilde{\psi}(a=1,d=0) - \tilde{\psi}(a=0,d=0)\} \\ &- \{\psi_0(a=1,d=0) - \psi_0(a=0,d=0)\}\end{aligned}$$

where

$$\begin{aligned}&\psi_0(a=1,d=0) - \psi_0(a=0,d=0) \\ &= \mu_0(1,1,1)p_L p_U + \mu_0(1,1,0)p_L(1-p_U) \\ &\quad + \mu_0(1,0,1)(1-p_L)p_U + \mu_0(1,0,0)(1-p_L)(1-p_U) \\ &\quad - \mu_0(0,1,1)p_L p_U - \mu_0(0,1,0)p_L(1-p_U) \\ &\quad - \mu_0(0,0,1)(1-p_L)p_U - \mu_0(0,0,0)(1-p_L)(1-p_U)\end{aligned} \tag{S7}$$

We next derive a suitable expression for $\tilde{\psi}(a=1,d=0) - \tilde{\psi}(a=0,d=0)$, where recall that $\tilde{\psi}(a,d=0) = \sum_l \Pr(Y=1|D=0,A=a,L=l)\Pr(L=l)$. To do so, it will be convenient to express $\mu(a,l) := \Pr(Y=1|D=0,A=a,L=l)$ in terms of $\mu_0(a,l,u)$. By standard probability laws and the independence assumptions encoded in the DAG,

$$\begin{aligned}\mu(a,l) &= \sum_u \Pr(Y=1|D=0,A=a,L=l,U=u)\Pr(U=u|D=0,A=a,L=l) \\ &= \sum_u \Pr(Y=1|D=0,A=a,L=l,U=u)\times \\ &\qquad \frac{\Pr(D=0|A=a,L=l,U=u)\Pr(L=l,U=u|A=a)}{\sum_u \Pr(D=0|A=a,L=l,U=u)\Pr(L=l,U=u|A=a)} \\ &= \frac{\sum_u \mu_0(a,l,u)\{1-\pi_0(a,l,u)\}\Pr(L=l,U=u)}{\sum_u \{1-\pi_0(a,l,u)\}\Pr(L=l,U=u)} \\ &= \frac{\sum_u \mu_0(a,l,u)\{1-\pi_0(a,l,u)\}\Pr(U=u)}{\sum_u \{1-\pi_0(a,l,u)\}\Pr(U=u)}\end{aligned} \tag{S8}$$

Plugging (S8) into the expression for $\tilde{\psi}(a, d=0)$,

$$
\begin{aligned}
&\tilde{\psi}(a=1, d=0) - \tilde{\psi}(a=0, d=0) \\
&= \frac{\mu_0(1,1,1)\{1-\pi_0(1,1,1)\}p_U + \mu_0(1,1,0)\{1-\pi_0(1,1,0)\}(1-p_U)}{\{1-\pi_0(1,1,1)\}p_U + \{1-\pi_0(1,1,0)\}(1-p_U)} p_L \\
&\quad + \frac{\mu_0(1,0,1)\{1-\pi_0(1,0,1)\}p_U + \mu_0(1,0,0)\{1-\pi_0(1,0,0)\}(1-p_U)}{\{1-\pi_0(1,0,1)\}p_U + \{1-\pi_0(1,0,0)\}(1-p_U)} (1-p_L) \\
&\quad - \frac{\mu_0(0,1,1)\{1-\pi_0(0,1,1)\}p_U + \mu_0(0,1,0)\{1-\pi_0(0,1,0)\}(1-p_U)}{\{1-\pi_0(0,1,1)\}p_U + \{1-\pi_0(0,1,0)\}(1-p_U)} p_L \\
&\quad - \frac{\mu_0(0,0,1)\{1-\pi_0(0,0,1)\}p_U + \mu_0(0,0,0)\{1-\pi_0(0,0,0)\}(1-p_U)}{\{1-\pi_0(0,0,1)\}p_U + \{1-\pi_0(0,0,0)\}(1-p_U)} (1-p_L)
\end{aligned}
$$

which can be re-expressed as

$$
\begin{aligned}
&\tilde{\psi}(a=1, d=0) - \tilde{\psi}(a=0, d=0) \\
&= \frac{1-\pi_0(1,1,1)}{\{1-\pi_0(1,1,1)\}p_U + \{1-\pi_0(1,1,0)\}(1-p_U)} \mu_0(1,1,1) p_L p_U \\
&\quad + \frac{1-\pi_0(1,1,0)}{\{1-\pi_0(1,1,1)\}p_U + \{1-\pi_0(1,1,0)\}(1-p_U)} \mu_0(1,1,0) p_L (1-p_U) \\
&\quad + \frac{1-\pi_0(1,0,1)}{\{1-\pi_0(1,0,1)\}p_U + \{1-\pi_0(1,0,0)\}(1-p_U)} \mu_0(1,0,1)(1-p_L) p_U \\
&\quad + \frac{1-\pi_0(1,0,0)}{\{1-\pi_0(1,0,1)\}p_U + \{1-\pi_0(1,0,0)\}(1-p_U)} \mu_0(1,0,0)(1-p_L)(1-p_U) \\
&\quad - \frac{1-\pi_0(0,1,1)}{\{1-\pi_0(0,1,1)\}p_U + \{1-\pi_0(0,1,0)\}(1-p_U)} \mu_0(0,1,1) p_L p_U \\
&\quad - \frac{1-\pi_0(0,1,0)}{\{1-\pi_0(0,1,1)\}p_U + \{1-\pi_0(0,1,0)\}(1-p_U)} \mu_0(0,1,0) p_L (1-p_U) \\
&\quad - \frac{1-\pi_0(0,0,1)}{\{1-\pi_0(0,0,1)\}p_U + \{1-\pi_0(0,0,0)\}(1-p_U)} \mu_0(0,0,1)(1-p_L) p_U \\
&\quad - \frac{1-\pi_0(0,0,0)}{\{1-\pi_0(0,0,1)\}p_U + \{1-\pi_0(0,0,0)\}(1-p_U)} \mu_0(0,0,0)(1-p_L)(1-p_U)
\end{aligned}
\tag{S9}
$$

Plugging (S7) and (S9) into the expression for $\{\text{CDE}_{obs} - \text{CDE}_0\}$,

$$
\begin{aligned}
&\{\text{CDE}_{obs} - \text{CDE}_0\} \\
&= \left[\frac{1-\pi_0(1,1,1)}{\{1-\pi_0(1,1,1)\}p_U + \{1-\pi_0(1,1,0)\}(1-p_U)} - 1\right]\mu_0(1,1,1)p_Lp_U \\
&\quad + \left[\frac{1-\pi_0(1,1,0)}{\{1-\pi_0(1,1,1)\}p_U + \{1-\pi_0(1,1,0)\}(1-p_U)} - 1\right]\mu_0(1,1,0)p_L(1-p_U) \\
&\quad + \left[\frac{1-\pi_0(1,0,1)}{\{1-\pi_0(1,0,1)\}p_U + \{1-\pi_0(1,0,0)\}(1-p_U)} - 1\right]\mu_0(1,0,1)(1-p_L)p_U \\
&\quad + \left[\frac{1-\pi_0(1,0,0)}{\{1-\pi_0(1,0,1)\}p_U + \{1-\pi_0(1,0,0)\}(1-p_U)} - 1\right]\mu_0(1,0,0)(1-p_L)(1-p_U) \\
&\quad - \left[\frac{1-\pi_0(0,1,1)}{\{1-\pi_0(0,1,1)\}p_U + \{1-\pi_0(0,1,0)\}(1-p_U)} - 1\right]\mu_0(0,1,1)p_Lp_U \\
&\quad - \left[\frac{1-\pi_0(0,1,0)}{\{1-\pi_0(0,1,1)\}p_U + \{1-\pi_0(0,1,0)\}(1-p_U)} - 1\right]\mu_0(0,1,0)p_L(1-p_U) \\
&\quad - \left[\frac{1-\pi_0(0,0,1)}{\{1-\pi_0(0,0,1)\}p_U + \{1-\pi_0(0,0,0)\}(1-p_U)} - 1\right]\mu_0(0,0,1)(1-p_L)p_U \\
&\quad - \left[\frac{1-\pi_0(0,0,0)}{\{1-\pi_0(0,0,1)\}p_U + \{1-\pi_0(0,0,0)\}(1-p_U)} - 1\right]\mu_0(0,0,0)(1-p_L)(1-p_U)
\end{aligned}
$$

Upon algebraic simplification,

$$
\begin{aligned}
&\{\mathrm{CDE}_{obs} - \mathrm{CDE}_0\} \\
&= \frac{-\{\pi_0(1,1,1) - \pi_0(1,1,0)\}(1-p_U)}{\{1-\pi_0(1,1,1)\}p_U + \{1-\pi_0(1,1,0)\}(1-p_U)} \mu_0(1,1,1) p_L p_U \\
&\quad + \frac{\{\pi_0(1,1,1) - \pi_0(1,1,0)\}p_U}{\{1-\pi_0(1,1,1)\}p_U + \{1-\pi_0(1,1,0)\}(1-p_U)} \mu_0(1,1,0) p_L (1-p_U) \\
&\quad + \frac{-\{\pi_0(1,0,1) - \pi_0(1,0,0)\}(1-p_U)}{\{1-\pi_0(1,0,1)\}p_U + \{1-\pi_0(1,0,0)\}(1-p_U)} \mu_0(1,0,1)(1-p_L) p_U \\
&\quad + \frac{\{\pi_0(1,0,1) - \pi_0(1,0,0)\}p_U}{\{1-\pi_0(1,0,1)\}p_U + \{1-\pi_0(1,0,0)\}(1-p_U)} \mu_0(1,0,0)(1-p_L)(1-p_U) \\
&\quad - \frac{-\{\pi_0(0,1,1) - \pi_0(0,1,0)\}(1-p_U)}{\{1-\pi_0(0,1,1)\}p_U + \{1-\pi_0(0,1,0)\}(1-p_U)} \mu_0(0,1,1) p_L p_U \\
&\quad - \frac{\{\pi_0(0,1,1) - \pi_0(0,1,0)\}p_U}{\{1-\pi_0(0,1,1)\}p_U + \{1-\pi_0(0,1,0)\}(1-p_U)} \mu_0(0,1,0) p_L (1-p_U) \\
&\quad - \frac{-\{\pi_0(0,0,1) - \pi_0(0,0,0)\}(1-p_U)}{\{1-\pi_0(0,0,1)\}p_U + \{1-\pi_0(0,0,0)\}(1-p_U)} \mu_0(0,0,1)(1-p_L) p_U \\
&\quad - \frac{\{\pi_0(0,0,1) - \pi_0(0,0,0)\}p_U}{\{1-\pi_0(0,0,1)\}p_U + \{1-\pi_0(0,0,0)\}(1-p_U)} \mu_0(0,0,0)(1-p_L)(1-p_U) \\
&= -\frac{\pi_0(1,1,1) - \pi_0(1,1,0)}{\{1-\pi_0(1,1,1)\}p_U + \{1-\pi_0(1,1,0)\}(1-p_U)} \{\mu_0(1,1,1) - \mu_0(1,1,0)\} p_L p_U (1-p_U) \\
&\quad - \frac{\pi_0(1,0,1) - \pi_0(1,0,0)}{\{1-\pi_0(1,0,1)\}p_U + \{1-\pi_0(1,0,0)\}(1-p_U)} \{\mu_0(1,0,1) - \mu_0(1,0,0)\}(1-p_L) p_U (1-p_U) \\
&\quad + \frac{\pi_0(0,1,1) - \pi_0(0,1,0)}{\{1-\pi_0(0,1,1)\}p_U + \{1-\pi_0(0,1,0)\}(1-p_U)} \{\mu_0(0,1,1) - \mu_0(0,1,0)\} p_L p_U (1-p_U) \\
&\quad + \frac{\pi_0(0,0,1) - \pi_0(0,0,0)}{\{1-\pi_0(0,0,1)\}p_U + \{1-\pi_0(0,0,0)\}(1-p_U)} \{\mu_0(0,0,1) - \mu_0(0,0,0)\}(1-p_L) p_U (1-p_U)
\end{aligned}
$$

Derivation of (S6)

We follow a similar approach as that used in the derivation of (S4). By definition, the non-identification error is given by

$$
\begin{aligned}
\{\mathrm{SDE}_{obs}^{a_D} - \mathrm{SDE}_0^{a_D}\} &= \{\tilde{\psi}(a_Y = 1, a_D) - \tilde{\psi}(a_Y = 0, a_D)\} \\
&\quad - \{\psi_0(a_Y = 1, a_D) - \psi_0(a_Y = 0, a_D)\}
\end{aligned}
$$

where

$$
\begin{aligned}
&\psi_0(a_Y = 1, a_D) - \psi_0(a_Y = 0, a_D) \\
&= \mu_0(1,1,1)\{1 - \pi_0(a_D, 1, 1)\}p_L p_U \\
&\quad + \mu_0(1,1,0)\{1 - \pi_0(a_D, 1, 0)\}p_L(1 - p_U) \\
&\quad + \mu_0(1,0,1)\{1 - \pi_0(a_D, 0, 1)\}(1 - p_L)p_U \\
&\quad + \mu_0(1,0,0)\{1 - \pi_0(a_D, 0, 0)\}(1 - p_L)(1 - p_U) \\
&\quad - \mu_0(0,1,1)\{1 - \pi_0(a_D, 1, 1)\}p_L p_U \\
&\quad - \mu_0(0,1,0)\{1 - \pi_0(a_D, 1, 0)\}p_L(1 - p_U) \\
&\quad - \mu_0(0,0,1)\{1 - \pi_0(a_D, 0, 1)\}(1 - p_L)p_U \\
&\quad - \mu_0(0,0,0)\{1 - \pi_0(a_D, 0, 0)\}(1 - p_L)(1 - p_U) \qquad \text{(S10)}
\end{aligned}
$$

We next derive a suitable expression for $\tilde{\psi}(a_Y = 1, a_D) - \tilde{\psi}(a_Y = 0, a_D)$, where recall that

$$
\tilde{\psi}(a_Y, a_D) = \sum_l \Pr(Y = 1|A = a_Y, D = 0, L = l) \Pr(D = 0|A = a_D, L = l) \Pr(L = l)
$$

To do so, it will be convenient to express $\pi(a, l) := \Pr(D = 1|A = a, L = l)$ in terms of $\pi_0(a, l, u)$. By standard probability laws and the independence assumptions encoded in the DAG,

$$
\begin{aligned}
\pi(a, l) &= \sum_u \Pr(D = 1|A = a, L = l, U = u) \Pr(U = u|A = a, L = l) \\
&= \sum_u \Pr(D = 1|A = a, L = l, U = u) \frac{\Pr(L = l, U = u|A = a)}{\sum_u \Pr(L = l, U = u|A = a)} \\
&= \frac{\sum_u \pi_0(a, l, u) \Pr(L = l, U = u)}{\sum_u \Pr(L = l, U = u)} \\
&= \sum_u \pi_0(a, l, u) \Pr(U = u) \qquad \text{(S11)}
\end{aligned}
$$

Plugging (S8) and (S11) into the expression for $\tilde{\psi}(a_Y, a_D)$,

$$\begin{aligned}
&\tilde{\psi}(a_Y = 1, a_D) - \tilde{\psi}(a_Y = 0, a_D) \\
&= \frac{\mu_0(1,1,1)\{1-\pi_0(1,1,1)\}p_U + \mu_0(1,1,0)\{1-\pi_0(1,1,0)\}(1-p_U)}{\{1-\pi_0(1,1,1)\}p_U + \{1-\pi_0(1,1,0)\}(1-p_U)} \\
&\qquad \times \left[\{1-\pi_0(a_D,1,1)\}p_U + \{1-\pi_0(a_D,1,0)\}(1-p_U)\right] p_L \\
&+ \frac{\mu_0(1,0,1)\{1-\pi_0(1,0,1)\}p_U + \mu_0(1,0,0)\{1-\pi_0(1,0,0)\}(1-p_U)}{\{1-\pi_0(1,0,1)\}p_U + \{1-\pi_0(1,0,0)\}(1-p_U)} \\
&\qquad \times \left[\{1-\pi_0(a_D,0,1)\}p_U + \{1-\pi_0(a_D,0,0)\}(1-p_U)\right] (1-p_L) \\
&- \frac{\mu_0(0,1,1)\{1-\pi_0(0,1,1)\}p_U + \mu_0(0,1,0)\{1-\pi_0(0,1,0)\}(1-p_U)}{\{1-\pi_0(0,1,1)\}p_U + \{1-\pi_0(0,1,0)\}(1-p_U)} \\
&\qquad \times \left[\{1-\pi_0(a_D,1,1)\}p_U + \{1-\pi_0(a_D,1,0)\}(1-p_U)\right] p_L \\
&- \frac{\mu_0(0,0,1)\{1-\pi_0(0,0,1)\}p_U + \mu_0(0,0,0)\{1-\pi_0(0,0,0)\}(1-p_U)}{\{1-\pi_0(0,0,1)\}p_U + \{1-\pi_0(0,0,0)\}(1-p_U)} \\
&\qquad \times \left[\{1-\pi_0(a_D,0,1)\}p_U + \{1-\pi_0(a_D,0,0)\}(1-p_U)\right] (1-p_L)
\end{aligned}$$

which can be re-expressed as

$$\begin{aligned}
&\tilde{\psi}(a_Y = 1, a_D) - \tilde{\psi}(a_Y = 0, a_D) \\
&= \frac{\{1-\pi_0(1,1,1)\}[\{1-\pi_0(a_D,1,1)\}p_U + \{1-\pi_0(a_D,1,0)\}(1-p_U)]}{\{1-\pi_0(1,1,1)\}p_U + \{1-\pi_0(1,1,0)\}(1-p_U)} \mu_0(1,1,1)p_L p_U \\
&+ \frac{\{1-\pi_0(1,1,0)\}[\{1-\pi_0(a_D,1,1)\}p_U + \{1-\pi_0(a_D,1,0)\}(1-p_U)]}{\{1-\pi_0(1,1,1)\}p_U + \{1-\pi_0(1,1,0)\}(1-p_U)} \mu_0(1,1,0)p_L(1-p_U) \\
&+ \frac{\{1-\pi_0(1,0,1)\}[\{1-\pi_0(a_D,0,1)\}p_U + \{1-\pi_0(a_D,0,0)\}(1-p_U)]}{\{1-\pi_0(1,0,1)\}p_U + \{1-\pi_0(1,0,0)\}(1-p_U)} \mu_0(1,0,1)(1-p_L)p_U \\
&+ \frac{\{1-\pi_0(1,0,0)\}[\{1-\pi_0(a_D,0,1)\}p_U + \{1-\pi_0(a_D,0,0)\}(1-p_U)]}{\{1-\pi_0(1,0,1)\}p_U + \{1-\pi_0(1,0,0)\}(1-p_U)} \mu_0(1,0,0)(1-p_L)(1-p_U) \\
&- \frac{\{1-\pi_0(0,1,1)\}[\{1-\pi_0(a_D,1,1)\}p_U + \{1-\pi_0(a_D,1,0)\}(1-p_U)]}{\{1-\pi_0(0,1,1)\}p_U + \{1-\pi_0(0,1,0)\}(1-p_U)} \mu_0(0,1,1)p_L p_U \\
&- \frac{\{1-\pi_0(0,1,0)\}[\{1-\pi_0(a_D,1,1)\}p_U + \{1-\pi_0(a_D,1,0)\}(1-p_U)]}{\{1-\pi_0(0,1,1)\}p_U + \{1-\pi_0(0,1,0)\}(1-p_U)} \mu_0(0,1,0)p_L(1-p_U) \\
&- \frac{\{1-\pi_0(0,0,1)\}[\{1-\pi_0(a_D,0,1)\}p_U + \{1-\pi_0(a_D,0,0)\}(1-p_U)]}{\{1-\pi_0(0,0,1)\}p_U + \{1-\pi_0(0,0,0)\}(1-p_U)} \mu_0(0,0,1)(1-p_L)p_U \\
&- \frac{\{1-\pi_0(0,0,0)\}[\{1-\pi_0(a_D,0,1)\}p_U + \{1-\pi_0(a_D,0,0)\}(1-p_U)]}{\{1-\pi_0(0,0,1)\}p_U + \{1-\pi_0(0,0,0)\}(1-p_U)} \mu_0(0,0,0)(1-p_L)(1-p_U)
\end{aligned} \tag{S12}$$

Plugging (S10) and (S12) into the expression for $\{\text{SDE}^{a_D}_{obs} - \text{SDE}^{a_D}_{0}\}$,

$$
\begin{aligned}
&\{\text{SDE}^{a_D}_{obs} - \text{SDE}^{a_D}_{0}\} \\
&= \left[\frac{\{1-\pi_0(1,1,1)\}[\{1-\pi_0(a_D,1,1)\}p_U + \{1-\pi_0(a_D,1,0)\}(1-p_U)]}{\{1-\pi_0(1,1,1)\}p_U + \{1-\pi_0(1,1,0)\}(1-p_U)} - \{1-\pi_0(a_D,1,1)\}\right] \\
&\quad \times \mu_0(1,1,1)p_L p_U \\
&\quad + \left[\frac{\{1-\pi_0(1,1,0)\}[\{1-\pi_0(a_D,1,1)\}p_U + \{1-\pi_0(a_D,1,0)\}(1-p_U)]}{\{1-\pi_0(1,1,1)\}p_U + \{1-\pi_0(1,1,0)\}(1-p_U)} - \{1-\pi_0(a_D,1,0)\}\right] \\
&\quad \times \mu_0(1,1,0)p_L(1-p_U) \\
&\quad + \left[\frac{\{1-\pi_0(1,0,1)\}[\{1-\pi_0(a_D,0,1)\}p_U + \{1-\pi_0(a_D,0,0)\}(1-p_U)]}{\{1-\pi_0(1,0,1)\}p_U + \{1-\pi_0(1,0,0)\}(1-p_U)} - \{1-\pi_0(a_D,0,1)\}\right] \\
&\quad \times \mu_0(1,0,1)(1-p_L)p_U \\
&\quad + \left[\frac{\{1-\pi_0(1,0,0)\}[\{1-\pi_0(a_D,0,1)\}p_U + \{1-\pi_0(a_D,0,0)\}(1-p_U)]}{\{1-\pi_0(1,0,1)\}p_U + \{1-\pi_0(1,0,0)\}(1-p_U)} - \{1-\pi_0(a_D,0,0)\}\right] \\
&\quad \times \mu_0(1,0,0)(1-p_L)(1-p_U) \\
&\quad - \left[\frac{\{1-\pi_0(0,1,1)\}[\{1-\pi_0(a_D,1,1)\}p_U + \{1-\pi_0(a_D,1,0)\}(1-p_U)]}{\{1-\pi_0(0,1,1)\}p_U + \{1-\pi_0(0,1,0)\}(1-p_U)} - \{1-\pi_0(a_D,1,1)\}\right] \\
&\quad \times \mu_0(0,1,1)p_L p_U \\
&\quad - \left[\frac{\{1-\pi_0(0,1,0)\}[\{1-\pi_0(a_D,1,1)\}p_U + \{1-\pi_0(a_D,1,0)\}(1-p_U)]}{\{1-\pi_0(0,1,1)\}p_U + \{1-\pi_0(0,1,0)\}(1-p_U)} - \{1-\pi_0(a_D,1,0)\}\right] \\
&\quad \times \mu_0(0,1,0)p_L(1-p_U) \\
&\quad - \left[\frac{\{1-\pi_0(0,0,1)\}[\{1-\pi_0(a_D,0,1)\}p_U + \{1-\pi_0(a_D,0,0)\}(1-p_U)]}{\{1-\pi_0(0,0,1)\}p_U + \{1-\pi_0(0,0,0)\}(1-p_U)} - \{1-\pi_0(a_D,0,1)\}\right] \\
&\quad \times \mu_0(0,0,1)(1-p_L)p_U \\
&\quad - \left[\frac{\{1-\pi_0(0,0,0)\}[\{1-\pi_0(a_D,0,1)\}p_U + \{1-\pi_0(a_D,0,0)\}(1-p_U)]}{\{1-\pi_0(0,0,1)\}p_U + \{1-\pi_0(0,0,0)\}(1-p_U)} - \{1-\pi_0(a_D,0,0)\}\right] \\
&\quad \times \mu_0(0,0,0)(1-p_L)(1-p_U)
\end{aligned}
$$

Upon algebraic simplification,

$$
\begin{aligned}
&\{\mathrm{SDE}^{a_D}_{obs} - \mathrm{SDE}^{a_D}_{0}\} \\
&= \frac{\{1-\pi_0(1,1,1)\}\{1-\pi_0(a_D,1,0)\} - \{1-\pi_0(a_D,1,1)\}\{1-\pi_0(1,1,0)\}}{\{1-\pi_0(1,1,1)\}p_U + \{1-\pi_0(1,1,0)\}(1-p_U)} \\
&\qquad \times \{\mu_0(1,1,1) - \mu_0(1,1,0)\}\, p_L p_U (1-p_U) \\
&\quad + \frac{\{1-\pi_0(1,0,1)\}\{1-\pi_0(a_D,0,0)\} - \{1-\pi_0(a_D,0,1)\}\{1-\pi_0(1,0,0)\}}{\{1-\pi_0(1,0,1)\}p_U + \{1-\pi_0(1,0,0)\}(1-p_U)} \\
&\qquad \times \{\mu_0(1,0,1) - \mu_0(1,0,0)\}\, (1-p_L) p_U (1-p_U) \\
&\quad - \frac{\{1-\pi_0(0,1,1)\}\{1-\pi_0(a_D,1,0)\} - \{1-\pi_0(a_D,1,1)\}\{1-\pi_0(0,1,0)\}}{\{1-\pi_0(0,1,1)\}p_U + \{1-\pi_0(0,1,0)\}(1-p_U)} \\
&\qquad \times \{\mu_0(0,1,1) - \mu_0(0,1,0)\}\, p_L p_U (1-p_U) \\
&\quad - \frac{\{1-\pi_0(0,0,1)\}\{1-\pi_0(a_D,0,0)\} - \{1-\pi_0(a_D,0,1)\}\{1-\pi_0(0,0,0)\}}{\{1-\pi_0(0,0,1)\}p_U + \{1-\pi_0(0,0,0)\}(1-p_U)} \\
&\qquad \times \{\mu_0(0,0,1) - \mu_0(0,0,0)\}\, (1-p_L) p_U (1-p_U)
\end{aligned}
$$

Section S2. Implications of the two weighted estimators under near violations of the positivity condition

We discussed that even in settings where the positivity condition for competing events (6) holds, near violations of positivity may occur, which can inflate the variance (15) relative to (19). To see this more explicitly, in our simple setting both estimators (12) and (16) can be written as

$$\hat{E}\left[Y \times W(A=1) | A=1\right] - \hat{E}\left[Y \times W(A=0) | A=0\right]. \tag{S13}$$

differing only by the form of the weight $W(A)$. For (12), $W(A) = \frac{I(D=0)}{\{1-\tilde{\pi}(A,L;\hat{\hat{\beta}})\}}$ while, for (16), $W(A) = \frac{\{1-\tilde{\pi}(a_D,L;\hat{\hat{\beta}})\}}{\{1-\tilde{\pi}(A,L;\hat{\hat{\beta}})\}}$. In the case of a near positivity violation for a particular joint stratum of A, L, an individual in this stratum who does *not* experience the competing event (an unusual individual by the premise of a near positivity violation in this stratum), will necessarily have an extremely large weight value $W(A)$ for the IPCW estimator (12) (the denominator will be close to zero while the numerator is, by definition, one). By contrast, even for such an "extreme" individual, the form of $W(A)$ for the estimator (16) is inherently

"stabilized" (the denominator will be close to zero but the numerator is something between 0 and 1 and may also be very close to zero). In turn, in this setting, we would expect the variance (15) to be larger than (19).

Section S3. Detailed descriptions of the dataset structure and estimators

In the estrogen randomized trial, individuals $i = 1, ..., n$ were randomly assigned to either estrogen therapy $A = 1$ or placebo $A = 0$. Let Y_k and D_k denote indicators of death due to prostate cancer and a competing event (death due to cardiovascular failure) by follow up month $k = 0, \ldots, K+1 = 50$, with $Y_0 = D_0 = 0$, indicating that all individuals are alive at baseline. To represent the history of a random variable, we use overbars, such as $\bar{Y}_k = (Y_1, Y_2, ..., Y_k)$. In this study no individual is lost to follow-up prior to 50 months such that, given no measurement error, we fully observe, $(L, A, \overline{Y}_{K+1}, \overline{D}_{K+1})$ where L are measured baseline covariates.

A controlled direct effect is defined as

$$\text{CDE}_{k,0} \equiv \psi_{k,0}(a=1, \bar{d}=0) - \psi_{k,0}(a=0, \bar{d}=0), \tag{S14}$$

where

$$\psi_{k,0}(a, \bar{d}=0) \equiv \Pr(Y_k^{a,\bar{d}=0}), \tag{S15}$$

denotes the counterfactual risk at k under a hypothetical intervention that eliminates competing events (Robins and Greenland, 1992). Young et al. (2020) showed that under certain conditions, the IPCW estimator for (S15) can be constructed as

$$\sum_{j=0}^{k-1} \frac{\sum_{i=1}^{n} Y_{j+1,i}(1-Y_{j,i})W_{j,i}(\hat{\eta})I(A_i=a)}{\sum_{i=1}^{n}(1-Y_{j,i})W_{j,i}(\hat{\eta})I(A_i=a)} \prod_{s=0}^{j-1}\left[1 - \frac{\sum_{i=1}^{n} Y_{s+1,i}(1-Y_{s,i})W_{s,i}(\hat{\eta})I(A_i=a)}{\sum_{i=1}^{n}(1-Y_{s,i})W_{s,i}(\hat{\eta})I(A_i=a)}\right], \tag{S16}$$

where

$$W_{k,i}(\hat{\eta}) = \frac{I(D_{k+1,i}=0)}{\prod_{j=0}^{k}[1-\Pr(D_{j+1}=1|\bar{D}_j=\bar{Y}_j=0, L_i, A=a; \hat{\eta})]}, \tag{S17}$$

where $\Pr(D_{k+1} = 1|\bar{D}_k = \bar{Y}_k = 0, L_i, A = a; \eta)$ denotes a parametric model for the competing event hazard indexed by parameter vector η, with $\hat{\eta}$ the MLE of η. The weight (S17) is an extension of the weight used in (12), generalized by taking products of the denominator over time.

Stensrud et al. (2022) defined the separable direct effect at k as

$$\text{SDE}_{k,0} \equiv \psi_{k,0}(a_Y = 1, a_D) - \psi_{k,0}(a_Y = 0, a_D), \tag{S18}$$

where

$$\psi_{k,0}(a_Y, a_D) \equiv \Pr(Y_k^{a_Y, a_D}) \tag{S19}$$

denotes the counterfactual risk at k under hypothetical interventions $(A_Y, A_D) = (a_Y, a_D)$. Stensrud et al. (2022) showed that, under certain conditions, the weighted estimator for (S19) is

$$\sum_{j=0}^{k-1} \frac{\sum_{i=1}^{n} Y_{j+1,i}(1 - Y_{j,i})(1 - D_{j+1,i}) W_{j,i}^{a_D}(\hat{\eta}) I(A_i = a_Y)}{\sum_{i=1}^{n} I(A_i = a_Y)} \tag{S20}$$

where

$$W_{k,i}^{a_D}(\hat{\eta}) = \frac{\prod_{j=0}^{k} [1 - \Pr(D_{j+1} = 1|\bar{D}_j = \bar{Y}_j = 0, L_i, A = a_D; \hat{\eta})]}{\prod_{j=0}^{k} [1 - \Pr(D_{j+1} = 1|\bar{D}_j = \bar{Y}_j = 0, L_i, A = a_Y; \hat{\eta})]}. \tag{S21}$$

Again, the weight is an extension of the weight used in (16), generalized by taking products of both the numerators and the denominators over time.

The model for the competing event hazard, $\Pr(D_{k+1} = 1|\bar{D}_k = \bar{Y}_k = 0, L = l, A = a; \eta)$, is a nuisance function shared by both weighted estimators above. We applied a pooled logistic model to estimate this nuisance function. Estimated coefficients and standard errors are shown in Supplemental Table 4. Details of this model are provided in the main text, Section 5.

Section S4. Approximations under rare-disease assumption

Assume that Y and D are rare. Then, the logistic models (20) and (21) can be approximated as

$$\mu_0(a,l,u;\theta) \approx \exp\{\theta_0+\theta_1 a+\theta_2 l+\theta_3 al+\theta_4 u+\theta_5 au+\theta_6 lu\}$$
$$\pi_0(a,l,u;\beta) \approx \exp\{\beta_0+\beta_1 a+\beta_2 l+\beta_3 al+\beta_4 u+\beta_5 au+\beta_6 lu\}$$

By plugging these models into the formulas derived in Section S1, we can get approximated versions or errors under the parametric assumption and rare-disease assumption. For example, the estimand error (S2) can be approximated as,

$$\begin{aligned}
&\{\exp(\theta_0+\theta_1+\theta_2+\theta_3+\theta_4+\theta_5+\theta_6)-\exp(\theta_0+\theta_2+\theta_4+\theta_6)\}\times\\
&\quad\exp(\beta_0+\beta_1 a_D+\beta_2+\beta_3 a_D+\beta_4+\beta_5 a_D+\beta_6)p_L p_U\\
&\quad+\{\exp(\theta_0+\theta_1+\theta_2+\theta_3)-\exp(\theta_0+\theta_2)\}\exp(\beta_0+\beta_1 a_D+\beta_2+\beta_3 a_D)p_L(1-p_U)\\
&\quad+\{\exp(\theta_0+\theta_1+\theta_4+\theta_5)-\exp(\theta_0+\theta_4)\}\exp(\beta_0+\beta_1 a_D+\beta_4+\beta_5 a_D)(1-p_L)p_U\\
&\quad+\{\exp(\theta_0+\theta_1)-\exp(\theta_0)\}\exp(\beta_0+\beta_1 a_D)(1-p_L)(1-p_U)\\
&=\{\exp(\theta_1+\theta_3+\theta_5)-1\}\exp(\theta_0+\theta_2+\theta_4+\theta_6+\beta_0+\beta_1 a_D+\beta_2+\beta_3 a_D+\beta_4+\beta_5 a_D+\beta_6)p_L p_U\\
&\quad+\{\exp(\theta_1+\theta_3)-1\}\exp(\theta_0+\theta_2+\beta_0+\beta_1 a_D+\beta_2+\beta_3 a_D)p_L(1-p_U)\\
&\quad+\{\exp(\theta_1+\theta_5)-1\}\exp(\theta_0+\theta_4+\beta_0+\beta_1 a_D+\beta_4+\beta_5 a_D)(1-p_L)p_U\\
&\quad+\{\exp(\theta_1)-1\}\exp(\theta_0+\beta_0+\beta_1 a_D)(1-p_L)(1-p_U)
\end{aligned}$$

Consider the ratio of (S10) to (S7), which presents the estimand error in multiplicative scale, rather than the difference (S7) − (S10) examined in (S1). Assume that $p_U = 0$, implying that $\{\text{CDE}_{obs} - \text{CDE}_0\} = \{\text{SDE}^{a_D}_{obs} - \text{SDE}^{a_D}_0\} = 0$. If (S7) is nonzero, under the same parametric assumption and rare-disease assumption, the ratio of (S10) to (S7) is approximated as,

$$1-\exp(\beta_0+\beta_1 a_D)\frac{\exp(\theta_2+\beta_2+\beta_3 a_D)\{\exp(\theta_1+\theta_3)-1\}p_L+\{\exp(\theta_1)-1\}(1-p_L)}{\exp(\theta_2)\{\exp(\theta_1+\theta_3)-1\}p_L+\{\exp(\theta_1)-1\}(1-p_L)}$$

This function can be negative for some parameter combinations (e.g., for $a_D = 1, p_L = 0.5$ and $(\theta_1,\theta_2,\theta_3,\beta_0,\beta_1,\beta_2,\beta_3) = (1, 1.5, -1.5, -6, 1, 1, 1)$). That is, (S7) and (S10), and in turn,

CDE_0 and $\mathrm{SDE}_0^{a_D}$ can have opposite signs.

References

Robins, J. M. and Greenland, S. (1992). Identifiability and exchangeability for direct and indirect effects. *Epidemiology* **3,** 143–155.

Stensrud, M. J., Young, J. G., Didelez, V., Robins, J. M., and Hernán, M. A. (2022). Separable effects for causal inference in the presence of competing events. *Journal of the American Statistical Association* **117,** 175–183.

Young, J. G., Stensrud, M. J., Tchetgen Tchetgen, E. J., and Hernán, M. A. (2020). A causal framework for classical statistical estimands in failure-time settings with competing events. *Statistics in Medicine* **39,** 1199–1236.

Supplemental Table 1: Ranges of parameter values for logistic models (20) and (21) in data generating scenarios for Figures 3, 4, and Supplemental Figure 1. The intercept θ_0 for the model (20) was fixed at -1 in all figures. The intercept β_0 for the model (21) was set to -1 for all panels in Figures 3 and 4 (where D is non-rare), and to -9 (left panels) and -6 (right panels) in Supplemental Figure 1 (where D is rare).

Parameter	Description	Range of values
θ_1	Dependence of Y on A	-1, -0.5, 0.5, 1
θ_2	Dependence of Y on L	-1, -0.5, 0.5, 1
θ_3	Dependence of Y on the interaction of (A, L)	-1, -0.5, 0.5, 1
θ_4	Dependence of Y on U	-1, -0.5, 0.5, 1
θ_5	Dependence of Y on the interaction of (A, U)	-1, -0.5, 0.5, 1
θ_6	Dependence of Y on the interaction of (L, U)	-1, -0.5, 0.5, 1
β_1	Dependence of D on A	-1, -0.5, 0.5, 1
β_2	Dependence of D on L	-1, -0.5, 0.5, 1
β_3	Dependence of D on the interaction of (A, L)	-1, -0.5, 0.5, 1
β_4	Dependence of D on U	-1, -0.5, 0.5, 1
β_5	Dependence of D on the interaction of (A, U)	-1, -0.5, 0.5, 1
β_6	Dependence of D on the interaction of (L, U)	-1, -0.5, 0.5, 1

Supplemental Table 2: Specifications of the logistic model coefficients used in simulation study, Section 4.3. In all scenarios, $(p_L, p_U, \theta_0, \theta_1, \theta_2, \theta_3)$ were fixed at $(0.1, 0.5, -1, -2, 1, 3)$

Near positivity violation?	Dependence of Y and D on U?	Competing events marginally rare?	θ_4	θ_5	θ_6	β_0	β_1	β_2	β_3	β_4	β_5	β_6
No	No	Yes	0	0	0	-3	1	1	-1	0	0	0
No	No	No	0	0	0	-1	1	1	-1	0	0	0
No	Yes	No	1	-2	0	-3	1	1	-1	3	1	0
Yes	No	Yes	0	0	0	-10	1	16	-1	0	0	0
Yes	No	No	0	0	0	-1	1	7	-1	0	0	0
Yes	Yes	No	1	-2	0	-3	1	7	-1	3	1	0

Supplemental Table 3: Simulation-based evaluation of the variance $\text{Var}(\widehat{\text{SDE}}_{obs}^{a_D=1})$. Expectations in the variance calculations were taken relative to the distribution over simulation runs.

Near positivity violation?	Dependence of Y and D on U?	Competing events marginally rare?	$\text{Var}(\widehat{\text{SDE}}_{obs}^{a_D=1})$
No	No	Yes	5.2×10^{-6}
No	No	No	2.5×10^{-6}
No	Yes	No	2.8×10^{-6}
Yes	No	Yes	4.5×10^{-6}
Yes	No	No	1.9×10^{-6}
Yes	Yes	No	2.2×10^{-6}

Supplemental Table 4: Regression coefficients for other-cause death by the pooled logistic model fitted to the prostate cancer data used in Section 5.

	Estimate	Std. Error
Intercept	-5.12	0.71
Month	-0.02	0.02
Month^2	0.00	0.00
Activity level (normal activity)	-0.28	0.30
Age (60-75)	0.70	0.60
Age ($\geq$ 75)	1.09	0.61
History of cardiovascular disease	0.33	0.30
Serum hemoglobin (<12 g/100ml)	0.60	0.24
Treatment (5.0 mg estrogen)	-0.14	0.31
Treatment $\times$ History of cardiovascular disease	0.76	0.41

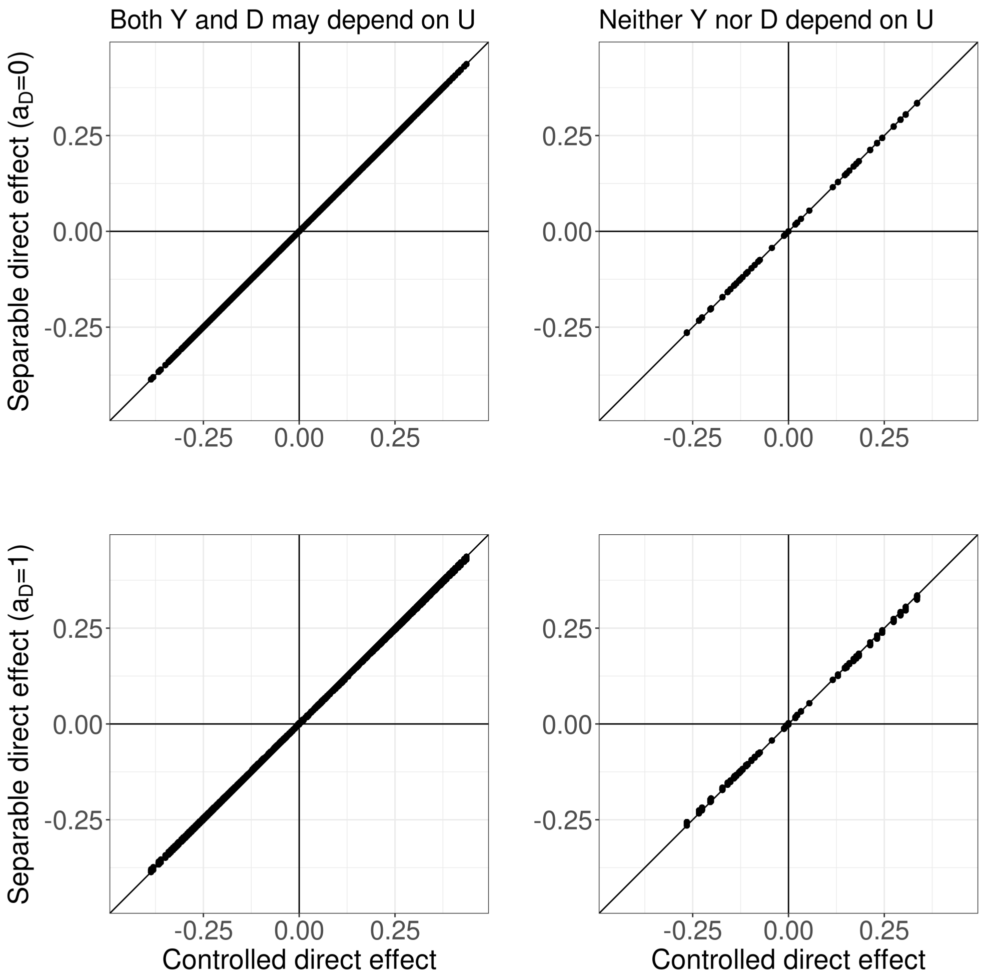

Supplemental Figure 1: Illustration of estimand error when the actual causal target is a separable direct effect but the ostensible causal target is a controlled direct effect in the scenarios where D is rare (i.e., under the parameters given in Supplemental Table 1 where $\Pr(D = 1|A = a, L = l, U = u) < 10\%$ for all (a, l, u)). Each dot plots the value of the actual versus the ostensible causal target for a combination of the parameter values.
